# Cardiogenic shock following administration of propofol and fentanyl in a healthy woman: a case report

**DOI:** 10.1186/1752-1947-5-382

**Published:** 2011-08-16

**Authors:** Alfredo Renilla González, Iñigo Lozano Martinez-Luengas, Sandra Secades González, Irene Álvarez Pichel, Paloma Álvarez Martinez, Elena Santamarta Liébana, Beatriz Díaz Molina

**Affiliations:** 1 Cardiology Department, Hospital Universitario Central de Asturias, Julián Claveria s/n 33005, Oviedo, Spain; 2 Radiology Department, Hospital Universitario Central de Asturias, Oviedo, Spain

## Abstract

**Introduction:**

Cardiogenic shock is very uncommon in healthy people. The differential diagnosis for patients with acute heart failure in previously healthy hearts includes acute myocardial infarction and myocarditis. However, many drugs can also depress myocardial function. Propofol and fentanyl are frequently used during different medical procedures. The cardiovascular depressive effect of both drugs has been well established, but the development of cardiogenic shock is very rare when these agents are used.

**Case presentation:**

After a minor surgical intervention, a 32-year-old Caucasian woman with no significant medical history went into sudden hemodynamic deterioration due to acute heart failure. An urgent echocardiogram showed severe biventricular dysfunction and an estimated left ventricular ejection fraction of 20%. Extracorporeal life support and mechanical ventilation were required. Five days later her ventricular function had fully recovered, which allowed the progressive withdrawal of medical treatment. Prior to her hospital discharge, cardiac MRI showed neither edema nor pathological deposits on the delayed contrast enhancement sequences. At her six-month follow-up examination, the patient was asymptomatic and did not require treatment.

**Conclusion:**

Although there are many causes of cardiogenic shock, the presence of abrupt hemodynamic deterioration and the absence of a clear cause could be related to the use of propofol and fentanyl.

## Introduction

Cardiogenic shock is the most serious form of presentation of heart failure (HF). The anticipation of hemodynamic deterioration and multiple organ failure in these patients is very important in terms of survival. The outcome for patients with refractory acute cardiogenic shock remains disproportionately poor. Technological advances in recent years have enabled the development of new treatments, such as extracorporeal life support (ELS). ELS is a variation of cardiopulmonary bypass which could improve the outcomes of patients in cardiogenic shock [1]. Although ischemic heart disease is the most common cause, there are many other etiologies [2]. Some drugs commonly used for sedation and analgesia during surgical procedures, as frequently as electrical cardioversion or transesophageal echocardiography, may have undesirable effects on cardiac hemodynamics. Propofol and fentanyl could depress myocardial function, but the effect of these agents on left ventricular ejection fraction (LVEF) in patients with normal left ventricle function has been reported to be mildly reduced [3,4]. The development of cardiogenic shock in patients treated with these drugs is a very uncommon situation.

## Case presentation

**Figure 1:**
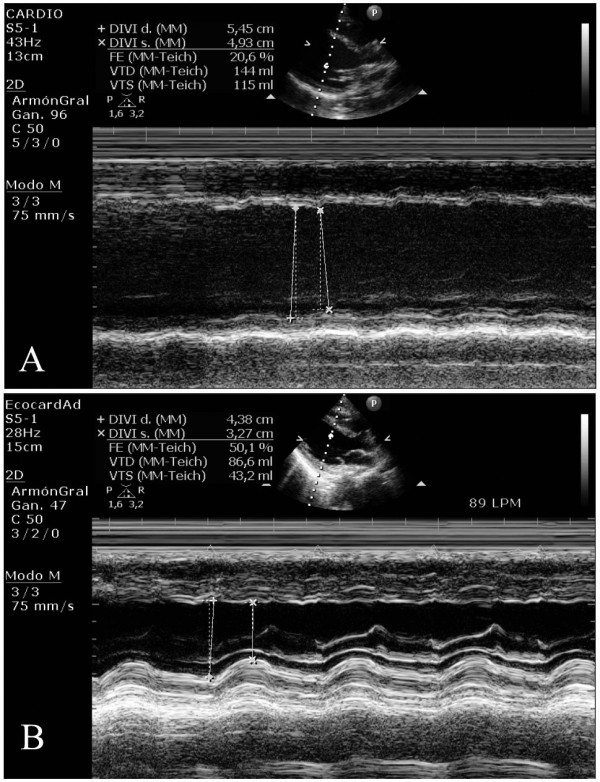
**Ecocardiographic images: (A)**  Transthoracic echocardiogram showing severe left ventricular dysfunction. **(B) **Normal LVEF after total recovery.

**Figure 2:**
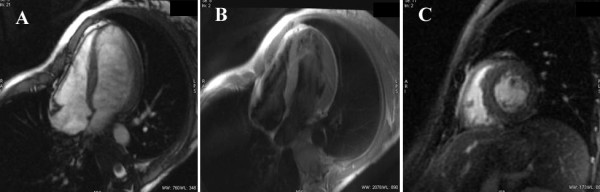
**Cardiac-MRI images: (A)** Cardiac MRI gradient echo sequence showing normal dimensions and function of the left ventricle. **(B)** T2-weighted short τ inversion recovery sequence showing the absence of edema. **(C)** Late gadolinium hyperenhancement sequence without pathological contrast captation.

We report the case of a 32-year-old Caucasian woman who experienced sudden, severe hemodynamic deterioration after undergoing a minor surgical procedure. Her medical history was unremarkable except for a vaginal delivery two years before. She underwent surgery to remove a Bartholin cyst, and no infection in the gland was found. The operation was performed while the patient was under sedation and being given an analgesic. Spontaneous breathing was maintained by infusing a propofol bolus (1 mg/kg) and fentanyl 100 μg intravenously. During surgery, the patient remained hemodynamically stable. She has nausea and vomiting in the early post-operative period, which were treated with intravenous ondansetron (4 mg). A few minutes later the patient went into sudden hemodynamic deterioration, with sinus tachycardia (113 regular beats/minute) and hypotension (50/30 mmHg). Pulse oximetry showed that her oxygen saturation level had decreased to 80% despite oxygen supplementation through a face mask (fraction of inspired oxygen 40%). In this clinical situation, we treated her with intravenous dopamine and dobutamine, as well as with mechanical ventilation because of global respiratory failure (arterial gasometry: oxygen pressure 40 mmHg, carbon dioxide pressure 49 mmHg). The electrocardiogram showed sinus tachycardia. Signs of HF were found on her chest X-ray, and urgent transthoracic echocardiography (TTE) revealed severe biventricular dysfunction with global hypokinesia and a LVEF estimated to be 35%. Coronary angiography showed no coronary lesions, and an intra-aortic balloon pump was inserted for counterpulsation. Repeat TTE revealed a LVEF of 20% with a dilated left ventricle (Figure 1A and Additional files 1 and 2, movies 1 and 2). Because of the patient's impaired clinical course, a left ventricular extracorporeal membrane oxygenation (ECMO) assistance device was inserted. After the fifth day, the patient's gradual recovery of LVEF led to the withdrawal of circulatory support and mechanical ventilation. Three weeks later a new TTE showed a non-dilated left ventricle, an absence of segmental contractility alterations, and a LVEF in the normal range (Figure 1B and Additional files 3 and 4, movies 3 and 4). The maximum value of troponin T was 0.60 ng/ml, and the C-reactive protein level was 6 mg/L. The patient's basic chemistry panel, complete blood cell count, and coagulation profile were within normal limits. The serology battery for myocarditis, blood cultures, urine cultures, and cytotoxic antibodies were all negative. An endomyocardial biopsy was not performed because of its low diagnostic yield. Prior to the patient's discharge, cardiac MRI was performed, which showed a preserved LVEF (Figure 2). Neither interstitial edema nor pathological deposits in the delayed enhancement sequences were seen. At her six-month follow-up examination, the patient was asymptomatic and did not require further treatment.

## Discussion

To establish the causal diagnosis of HF, it is necessary to determine whether the clinical presentation is a *de novo *process or a chronic entity exacerbated by surgery. In our patient, the absence of ventricular remodeling visualized by TTE suggests the former postulate. Regarding its etiology, many possibilities should be taken into account. Post-partum cardiomyopathy usually develops in late pregnancy or during the first months after delivery [5]. In our patient, childbirth was very unlikely the cause of her acute HF as delivery had occurred two years before. A viral infection could justify the clinical context of acute myocarditis [6], but her sudden clinical deterioration, with no history of infection or negative serologies and lack of typical findings on MRI, makes this diagnosis unlikely. Propofol infusion syndrome includes arrhythmias, hemodynamic deterioration, metabolic acidosis, rhabdomyolysis, and impaired renal and hepatic function. This clinical entity has been described mainly in pediatric critical care patients and has been associated with prolonged use (>48 hours) and high doses (>4 mg/kg/hour) [7]. Ondansetron is a 5-hydroxytryptamine type 3 (5-HT3) receptor antagonist used mainly as an anti-emetic. Although considered a safe class of medications by many clinicians, several of the 5-HT3 receptor antagonists have been associated with adverse cardiovascular effects [8]. There is a rare possibility of convulsions, chest pain, arrhythmias, hypotension, or bradycardia associated with using ondansetron, but we have not found any case in the literature describing a connection between the use of this drug in the post-operative and the development of HF. Takotsubo cardiomyopathy (TTC) is an acute cardiac syndrome mimicking elevated ST-segment myocardial infarction characterized by transient regional wall motion abnormalities involving the apical and middle portions of the left ventricle in the absence of significant obstructive coronary disease [9]. Recently, an apical sparing variant defined as akinesia of the basal and middle segments of all walls has been described [10]. In our patient, the absence of electrocardiographic and echocardiographic alterations suggestive of TTC leads us to reject this diagnostic possibility. The association of propofol and fentanyl as a cause of severe, acute HF has been described previously [11]. Other than the case described by Chow *et al*. [11], however, we have not found another case report in the literature that mentions the combination of these drugs as a cause of severe, acute HF due to ventricular dysfunction in patients with healthy hearts. In this regard, both propofol and fentanyl may cause depression of ventricular function and decreased blood pressure. Propofol dilates the arteries by inducing nitric oxide synthesis, blocks calcium channels, and activates protein kinase C, all of which, taken together, lead to a decrease in pre-load and a decline in cardiac output. Apart from this possibility, an intrinsic negative inotropic effect attributable to propofol itself has also been reported [4,12]. This effect is dose-dependent [13]. It occurs most often when used in combination with fentanyl and in patients with or without previous heart disease [4]. Both mechanisms might trigger a state of cardiogenic shock in patients with individual susceptibility. When these agents are used in combination, additional precautions should be taken in all patients, including those with normal left ventricular function. Because of refractory cardiogenic shock, ELS was needed in our patient. ELS should be considered in patients with severe, life-threatening respiratory or cardiac failure that does not respond to conventional intensive care management [1]. Currently, several options are available for circulatory support, including surgically implanted ventricular assistance devices, percutaneous assistance devices, and ECMO [14]. In our case, ECMO provided reasonable short-term support, allowing the patient to recover from multi-organ injury and increasing the time to complete a transplant evaluation if necessary. The use of this device is a support modality rather than a treatment in itself. As it requires well-trained personnel and is not without risk, selection of patients in whom this device can be used is required. So, the disease process must be reversible, or, failing this, the patient should be a candidate for transplantation or insertion of a ventricular assistance device.

## Conclusions

In conclusion, the final etiological diagnosis of our patient is uncertain. Her severe, acute hemodynamic deterioration due to acute heart failure seems to have been causally related to some event that occurred during the peri-operative period. Propofol and fentanyl are often used during different medical procedures. The effects previously described, although uncommon, should be taken into account in cases of abrupt hemodynamic deterioration and an absence of other possible causes. ECMO is an effective salvage strategy for the treatment of patients with extreme hemodynamic instability and multi-organ injury due to acute HF.

## Consent

Written informed consent was obtained from the patient for publication of this case report and any accompanying images. A copy of the written consent is available for review by the Editor-in-Chief of this journal.

## Competing interests

The authors declare that they have no competing interests.

## Authors' contributions

All authors read and approved the final manuscript.

## Supplementary Material

Additional file 1 **Apical view image showing severe left ventricular dysfunction**. Transthoracic echocardiography (apical four-chamber view) showing severe left ventricular dysfunction during the acute phase. Click here for file

Additional file 2 **Short-axis view showing severe left ventricular dysfunction**. Transthoracic echocardiography (paraesternal short-axis view) showing severe left ventricular dysfunction during the acute phase. Click here for file

Additional file 3 **Apical four-chamber view after total recovery of left ventricular function**. Transthoracic echocardiography (apical four-chamber view) showing total recovery of left ventricular function before discharge. Click here for file

Additional file 4 **Short-axis view after total recovery of left ventricular function**. Transthoracic echocardiography (paraesternal short-axis view) showing total recovery of left ventricular function before discharge. Click here for file
